# Supplementary material for: A single Citrobacter rodentium infection in Pink1 knockout and wild-type mice leads to regional blood-brain-barrier perturbation and limited microglial activation without dopamine neuron axon terminal loss
Source: PLoS Pathog. 2026 Jun 30;22(6):e1014315. doi: 10.1371/journal.ppat.1014315 (PMC13318054; doi:10.1371/journal.ppat.1014315)
Supplement: Attachment — Submitted filename: Response_reviewers_Trudeau_Plos_Pathogens.pdf [file ppat.1014315.s009.pdf]

Response to the reviewers

We thank the reviewers for their thoughtful and constructive comments, which have helped improve the clarity and interpretation of our study.

In response to the reviewers' major concerns, we have added CD68-based microglial activation analysis, quantified nigral and VTA dopaminergic neuron populations, incorporated RNA-seq analysis of tight junction related genes, clarified methods and experimental rationale and highlighted the major limitations of our study. Where additional experiments were not feasible, we strengthened the manuscript through more cautious interpretation. We also added a final schematic diagram (new figure 13) to summarize the systemic and central effects of *Citrobacter rodentium* infection that we observed in Pink1 WT and KO mice. All changes to the text are identified in yellow highlighting.

Below we provide a response to each comment and describe the corresponding revisions made to the manuscript.

Reviewer #1: As noted, the title suggests glial activation is a significant outcome from infection and modulated by Pink1, however, the actual microglial activation data is quite superficial, showing only western blot increase in total Iba1**,** and no morphological changes in the striatum. It was not stated why CD68 was not used as a marker for microglial activation, but this is routinely used as an indicator along with morphology to determine activation status of microglia – could the authors show this as a separate analysis to understand why the western data seem to contradict the imaging? As a minor note, as astrocytes were not activated, a better description would be microglial activation and not "glial" activation when discussing this result.

Response: We performed additional Iba1/CD68 double labelling to address this concern. The results are shown in the **new supplementary figure 2** and in the revised manuscript on page 12. So, globally, we observed a quantifiable increase in IBA1 levels in striatal lysates in infected Pink1 KO mice, whereas quantitative immunofluorescence did not detect changes in microglial density or morphology or number and CD68 immunoreactivity remained unchanged (**Figure S2, supplementary material**). These findings are not necessarily contradictory. IBA1 upregulation can reflect a mildly activated myeloid state and might not be specific to highly phagocytic microglia, whereas CD68 more closely reports lysosomal/phagocytic (glycosylated lysosomal protein) activation. In addition, western blot integrates signal across the full dissected tissue and can capture modest contributions from non-parenchymal myeloid compartments that may not be evident in brain parenchyma-focused morphological analyses. Consistent with preserved dopaminergic terminals and intact SNc/VTA neuron numbers at day 26, our data support the hypothesis that in the present infection model, there is modest myeloid activation rather than phagocytic microglial remodeling.

Reviewer #1: Given the focus on this link to PD, it is notable that striatal terminals were the only indicator of dopaminergic dysfunction reported. For the answer if Pink1 mediated response to infection on the DA system, why were nigral DA neurons not included in any analyses? Presumably if the immune/Pink1/mitochondrial interactions that have been previously reported were involved, mitochondrial damage in DA neurons might be a predegenerative response to this type of injury. Related, could nigral microglia have a different activation status than striatum if more damage is related to intracellular changes in mitochondria in DA neurons? Likewise, neurochemistry in the striatum could have potentially been a more sensitive measure (e.g., DA and metabolite levels), than DAT and TH loss.

Response:
We thank the reviewer for this important suggestion. To determine whether the increased BBB permeability and neuroinflammatory responses observed following *Citrobacter rodentium* infection were associated with dopaminergic neurodegeneration, we quantified tdTomato-positive neurons in the substantia nigra pars compacta (SNc) and ventral tegmental area (VTA) using automated image analysis. No significant differences in dopaminergic neuron numbers were detected between control and infected animals in either WT or Pink1 KO mice. These findings indicate that infection-induced BBB dysfunction and microglial activation occur in the absence of dopaminergic neuron loss at day 26 post-infection. This supports the interpretation that vascular and inflammatory alterations represent early events that precede neurodegeneration rather than direct consequences of neuronal death. We have incorporated these new data into the **new figure 8** and they are described in the revised manuscript on page 11.

We also acknowledge that striatal dopamine neurochemistry (e.g., dopamine and metabolite levels) could represent a more sensitive early indicator of neuronal dysfunction than DAT or TH loss, as functional alterations often precede structural degeneration. However, the present study was designed to assess infection induced BBB permeability, neuroimmune responses, and dopaminergic neuron integrity rather than neurotransmitter dynamics. The preservation of dopaminergic terminals and neuron numbers support the interpretation that observed vascular and inflammatory changes occur at an early, pre-neurodegenerative stage. Hence, we did not conduct any neurochemistry analysis. But we plan to do this in the near future in a separate study.

Reviewer 1#: The MRI data demonstrate potential changes in BBB but the ZO-1 and ZO-1 western blots suggest this is not related to endothelial tight junctions. While this certainly could be true, why are other BBB proteins not measured to determine what may lead to permeability? Immunohistochemistry of the brain vasculature would be helpful to demonstrate if regional differences on MRI are related to the reported neuro-immune response and modulation by Pink1.

Response: We respectfully emphasize that ZO-1 (TJP1) and ZO-2 (TJP2) are central and indispensable scaffolding proteins of the tight junction complex and are widely regarded as key

indicators of tight junction integrity in the blood brain barrier. ZO proteins physically link transmembrane tight junction components, including claudins and occludin, to the actin cytoskeleton and are required for tight junction assembly, maintenance, and barrier function. The absence of significant changes in either ZO-1 or ZO-2 across genotypes and treatment conditions indicates that major structural disorganization of the tight junction complex is unlikely to underlie the modest BBB permeability changes detected by MRI at this time point.

But we also agree with the reviewer that additional protein-level validation of tight junction markers would provide further mechanistic insight into BBB alterations. While these experiments could not be performed within the timeline allocated for revision, we leveraged our existing transcriptomic dataset to assess the expression of multiple tight junction related genes using normalized read counts prior to differential expression (DEG) analysis. Specifically, we examined canonical tight junction scaffolding and transmembrane components, including Tjp3, Ocln, Cldn5, Cldn12, Cldn3 and Cdh5. Statistical comparisons between infected wild type and infected Pink1 knockout mice were performed for all genes shown in the **new supplementary figure 6** using unpaired t-tests with Welch's correction. The results are described in the revised manuscript on page 15. None of the junctional genes exhibited significant differences between the two groups (all $p > 0.05$). These RNA-seq findings are consistent with our protein level analyses of ZO-1 and ZO-2 and collectively indicate that there is no evidence of major transcriptional or structural disruption of core BBB junctional components at day 26 post infection. This supports the interpretation that the modest BBB permeability changes observed by MRI are likely mediated by functional or regulatory mechanisms rather than breakdown of tight junction architecture.

Reviewer #2: One of the main points that needs clarification is why the authors specifically focused on ZO-1 and ZO-2 and did not test additional tight junction or adherens proteins. Relying on just two markers feels insufficient to support the conclusion that the BBB is not compromised. Later in the study bulk transcriptomic analysis is performed on the striatum, but since this was not restricted to endothelial cells, the results may also reflect expression from other cell types such as the ventricular lining (which will be isolated with the striatum). It would strengthen the manuscript if some of the tight junction or adherens markers identified from the transcriptomic dataset could be validated at the protein level, for example by western blot or immunohistochemistry, to support the overall conclusions.

Response: Please see our reply to the third comment of reviewer 1.

Reviewer #2: The study also addresses microglial reactivity in the striatum, using both western blot and immunofluorescence. The results are not consistent: there is an increase in Iba-1 in lysates, but no detectable difference in the tissue sections. Since Iba-1 is not specific to microglia—it is also expressed by perivascular macrophages, infiltrating monocytes, and macrophages in the choroid plexus (which could also be isolated with the striatum) -signal detected in western blots could be influenced by these populations. The flow cytometry results show that inflammatory

monocytes are present, even if not significantly altered, which complicates interpretation. Because the immunohistochemistry data are spatially restricted to the striatum, I would place more weight on those results. At present, the increase in Iba-1 in lysates does not provide strong enough evidence for microglial reactivity, and the conclusions in this section should be revised accordingly.

Response: We thank the reviewer for this important point and agree that interpretation of Iba1 increases in striatal lysates must be made cautiously. As noted, Iba1 is expressed not only by parenchymal microglia but also by other myeloid populations (e.g., perivascular macrophages and infiltrating monocytes), and bulk lysate measurements cannot localize the cellular source of the signal. In addition, our flow cytometry data indicate the presence of inflammatory monocytes in brain homogenates, which could contribute to IBA1 signal in western blot. Therefore, we expanded our immunofluorescence analysis by including CD68 together with Iba1 in striatal sections. CD68 is commonly used to assess lysosomal/phagocytic activation associated with reactive microglia. In this analysis, we did not detect a significant change in CD68-positive signal (intensity) between control and infection groups, consistent with the absence of morphological remodeling or density changes in IBA1 positive cells. These new results are presented in **the new supplementary figure 2** and on page 12 of the revised manuscript. Accordingly, we have revised the results and discussion to place greater weight on the histological quantification and to moderate our conclusions. We now state that the western blot findings of a modest increase in IBA1 in striatal lysates may possibly reflect myeloid changes and contributions from non-parenchymal myeloid compartments, rather than a definitive microglial reactivity. The manuscript has been edited throughout to avoid overstatement.

Reviewer #2: The flow cytometry data also reveal an increase in neutrophils in the brain. However, the evidence presented does not distinguish whether these cells extravasate into the parenchyma or remain confined to the vasculature. Without histological confirmation, an increase in neutrophils by flow cytometry is not sufficient to conclude that they infiltrate the brain tissue.

Response: We agree with the reviewer that flow cytometry alone does not inherently distinguish between immune cells confined to the vasculature and those that have extravasated into the brain parenchyma. This is why, as stated in the original version of the present manuscript, we performed an *in vivo* labeling approach prior to tissue collection by intravenously injecting a fluorophore-conjugated (BV711) CD45 antibody to label circulating and vasculature associated immune cells. During subsequent flow cytometric analysis of immune cells from the brain, the CD45 BV711 positive population corresponding to intravascular or peripheral immune cells was excluded from the analysis. This gating strategy allowed us to selectively analyze cells that had entered the brain tissue. This is described in our material and method section (page 32).

Reviewer #2: Finally, I would recommend revising the discussion to tone down some of the conclusions. For instance, the statement that Citrobacter infection perturbs BBB integrity, facilitates entry of immune cells, and initiates or exacerbates PD-related pathology feels too strong

based on the data presented here (lines 642 - 645). Aside from MRI, there is little direct evidence of BBB breakdown, and no histological data showing infiltration of T cells or neutrophils into the brain parenchyma. The discussion would benefit from a more cautious interpretation that reflects the limitations of the dataset. Altogether, the title should also be adjusted so that it better represents the study's overall findings.

Response: We thank the reviewer for this important feedback and agree that some of the original statements in the discussion overstated the conclusions. We have now carefully revised the discussion (pages 16 to 23) with a more cautious tone, particularly in the section referring to BBB integrity, immune cell entry, and potential exacerbation of Parkinson's disease-related pathology. Specifically, we now describe the MRI findings as indicative of increased BBB permeability rather than significant BBB breakdown. We also explicitly acknowledge the absence of histological confirmation of T cell or neutrophil extravasation and discuss this as a limitation of the current study. The revised text emphasizes that the observed vascular and neuroimmune changes are modest and may represent early or transient perturbations rather than large scale barrier disruption. In line with this interpretation, we have also revised the manuscript title to more accurately reflect the study's findings, focusing on infection-associated BBB permeability changes and neuroimmune responses without implying direct initiation or exacerbation of neurodegenerative pathology.

Reviewer #3: The manuscript is an assembly of many different investigative methods, which at times feels somewhat disconnected given the choice of genotype. Where does one expect a difference based on DAT-dependent change in PINK1 gene expression?

Related to that, the reader doesn't easily understand why the team chose a DAT-cre-dependent cKO model for PINK1 deficiency, in particular without the authors explaining where in all their tissues DAT is expressed; by inference, when is PINK1 normally expressed in the cells of their target organs? The field has seen a very restricted and context-dependent pattern of (mitochondrial) PINK1 function.

Response: We would like to bring to the attention of the reviewer that we did not adopt a conditional gene deletion approach. Constitutive Pink1 KO mice were crossed with DAT-Ires-Cre mice to obtain mice with constitutive KO of Pink1 in all cells, but tdTomato expression in dopamine neurons. This is simply an approach to better visualize the dopamine system in some experiments. This was used in our experiment to quantify the number of dopamine neurons. The mouse breeding strategy is described in the methods section (page 24).

Reviewer #3: Related to that, one misses the precise definition of the WT genotype. How were the animals bred, were some pairings done with heterozygotes?

Response: WT mice correspond to littermates including one DAT-Ires-Cre allele and the two WT Pink1 alleles. The mouse breeding strategy is described in the methods section (page 24).

Reviewer #3: In what context was the tomato reporter protein used, why was it never shown? It would have easily provided some answers to point #2 made above.

Response: The tdTomato reporter was used to facilitate the identification and quantification of dopaminergic neurons in the substantia nigra pars compacta and ventral tegmental area. We now include the quantification of this signal in the **new figure 8**, allowing the quantification of tdTomato-positive neurons in both regions, demonstrating preserved dopaminergic neuron numbers following infection. These results are described on page 11 of the revised manuscript.

Reviewer #3: Did any adverse event / mortality occur in any infected mice?

Response: No mortality or severe adverse events were observed in any experimental group following *Citrobacter rodentium* infection. This information has now been explicitly stated in the methods (page 25).

Reviewer #3: Was there a sex effect in any readouts?

Response: The number of animals per group for each experimental assay was limited, as multiple experimental approaches were performed in parallel. Consequently, the study was not sufficiently powered to allow reliable statistical analysis of sex specific effects.

Reviewer #3: The real money one could argue lies in the transcriptome analysis given the choice in genotypic construct and comparison. Did the authors consider carrying out transcriptomics in the spleen homogenates?

Response: We agree that peripheral transcriptomic profiling would provide valuable complementary insight into systemic immune responses. However, the experimental design did not plan for the collection of fresh spleen to perform transcriptomics.

Reviewer #3: Did the authors attempt to validate any mRNA change identified, such as by qPCR or Western blotting? I think validation steps would greatly enhance the relevance of their findings.
Response: While additional validation would strengthen transcriptomic findings, further experimental validation was not feasible within the revision timeframe. To address this concern, we performed targeted analyses of BBB-related tight junction genes using normalized RNA-seq read counts prior to differential expression analysis. These analyses revealed no significant

transcriptional changes and are now included in the supplementary section **(Fig. S6)** and are mentioned in the revised results section (page 15).

Reviewer #3: Did the authors make any comparison in their readouts and findings from the single (here) vs 4 exposure (published) events with the pathogen, whether it be in the WT or DAT-cre cKO animals, and whether it be by mRNA change, Iba1 signal change, MRI analysis or FACS analyses of the spleen? This, to answer which transient changes may be of greatest relevance as a potential biomarker when one considers a more sustained (or reoccurring) 2nd hit, ie, the C. rodentium microbe exposure.

Response: We thank the reviewer for this insightful suggestion. We agree that a direct comparison between single and repeated exposure paradigms would be highly informative for distinguishing transient versus sustained pathological changes and for identifying potential biomarkers relevant to recurrent inflammatory insults. While such comparative analyses were not performed in the present study, we have now incorporated this point into the discussion (page 23), highlighting the importance of future studies examining multiple exposure models across molecular, cellular, imaging, and immune readouts in both WT and Pink1 KO mice.

Reviewer #3: I think the Discussion section should address some of the shortcomings / limitations of their study.

Response: We have substantially expanded the discussion to explicitly address several limitations, including the subtle nature of BBB permeability changes detected primarily by MRI, the absence of histological confirmation of immune cell infiltration and the inability to perform additional protein/RNA level validation experiments. These additions provide a more cautious interpretation of the findings.

Editorial and Minor Issues

We have addressed all minor comments including:

- Clarifying spleen enlargement interpretation to match raw data
- Defining acronyms at first use
- Improving figure readability and scaling
- Increasing font sizes where needed
