# Supplementary material for: A single Citrobacter rodentium infection in Pink1 knockout and wild-type mice leads to regional blood-brain-barrier perturbation and limited microglial activation without dopamine neuron axon terminal loss
Source: PLoS Pathog. 2026 Jun 30;22(6):e1014315. doi: 10.1371/journal.ppat.1014315 (PMC13318054; doi:10.1371/journal.ppat.1014315)
Supplement: Attachment — Submitted filename: Response_reviewers_Trudeau_Plos_Pathogens_May15_2026.pdf [file ppat.1014315.s010.pdf]

Response to the reviewers

As requested by reviewer #2, we modified the short title to better reflect the findings of the study. The new short title is: "Gut infection perturbs BBB but fails to cause dopamine terminal loss in Pink1 KO mice".

As requested by reviewer #3, we modified the methods section to state explicitly that the present study included both male and female mice, but that the study was underpowered for a sex analysis.

Other issues

We added links to our protocols, now available on protocols.io.

We added RRIDs to identify the source of all key reagents.

We added a Zenodo link in the Data and materials availability section to make available a Key Resource Table providing access to all primary data.
