# Supplementary material for: A single Citrobacter rodentium infection in Pink1 knockout and wild-type mice leads to regional blood-brain-barrier perturbation and limited microglial activation without dopamine neuron axon terminal loss
Source: PLoS Pathog. 2026 Jun 30;22(6):e1014315. doi: 10.1371/journal.ppat.1014315 (PMC13318054; doi:10.1371/journal.ppat.1014315)
Supplement: Peer review history

# Peer review: Decision Letter 0

**DOI:** 10.1371/journal.ppat.1014315.r001

**Authors:** D. Scott Samuels, Amanda L. Woerman

28 Aug 2025

PPATHOGENS-D-25-01667

A single Citrobacter rodentium infection in Pink1 knockout and wild type mice leads to regional blood-brain-barrier perturbation and glial activation without dopamine neuron axon terminal loss

PLOS Pathogens

Dear Dr. Trudeau,

Thank you for submitting your manuscript to PLOS Pathogens. Your manuscript was evaluated by members of the editorial board and three external referees. All of us agree that the line of investigation is interesting and important, but that more rigorous approaches to sample analysis are needed (see below). With new datasets as suggested in hand, you should reassess the title and discussion to ensure you are not overstating your findings.

We invite you to submit a substantially revised version of the manuscript that addresses all of the points raised during the review process. In particular, Reviewer 1 notes that the Western blot for Iba1 is neither the most rigorous nor broadly accepted approach for characterizing changes in microglia; the CD68 antibody should be incorporated, and a morphological assessment needs to be included. The assessment on dopaminergic neurons was limited to striatal terminals; omitting analysis of nigral dopaminegic terminals leaves a major question unaddressed given the selective vulnerability with this brain region in PD. In addition, there are also concerns about limiting BBB analysis to ZO-1 and ZO-2, which is a very limited assessment of endothelial tight junction integrity. Lastly, the transcriptomics dataset is something the reviewers were excited about, but they pointed out several limitations where you could have used these data better to help delineate the immune-driven changes in the mice.

Please submit your revised manuscript within 60 days Oct 27 2025 11:59PM. If you will need more time than this to complete your revisions, please reply to this message or contact the journal office at plospathogens@plos.org. When you're ready to submit your revision, log on to https://www.editorialmanager.com/ppathogens/ and select the 'Submissions Needing Revision' folder to locate your manuscript file.

Please include the following items when submitting your revised manuscript:

* A rebuttal letter that responds to each point raised by the editor and reviewer(s). You should upload this letter as a separate file labeled 'Response to Reviewers'. This file does not need to include responses to any formatting updates and technical items listed in the 'Journal Requirements' section below.

* A marked-up copy of your manuscript that highlights changes made to the original version. You should upload this as a separate file labeled 'Revised Manuscript with Track Changes'.

* An unmarked version of your revised paper without tracked changes. You should upload this as a separate file labeled 'Manuscript'.

If you would like to make changes to your financial disclosure, competing interests statement, or data availability statement, please make these updates within the submission form at the time of resubmission. Guidelines for resubmitting your figure files are available below the reviewer comments at the end of this letter.

We look forward to receiving your revised manuscript.

Kind regards,

Amanda L. Woerman

Academic Editor

PLOS Pathogens

D. Scott Samuels

Section Editor

PLOS Pathogens Sumita Bhaduri-McIntosh

Editor-in-Chief

PLOS Pathogens

orcid.org/0000-0003-2946-9497

Michael Malim

Editor-in-Chief

PLOS Pathogens

orcid.org/0000-0002-7699-2064

**Journal Requirements:**

1) Please ensure that the CRediT author contributions listed for every co-author are completed accurately and in full.

At this stage, the following Authors/Authors require contributions: Sriparna Mukherjee, Vladimir Grouza, Alex Tchung, Amandine Even, Moein Yaqubi, Marius Tuznik, Tyler Canon, Sherilyn Junelle Recinto, Christina Gavino, Marie-Josée Bourque, Heidi McBride, Michel Desjardins, Samantha Gruenheid, Jo Anne Stratton, David A. Rudko, and Louis-Eric Trudeau. Please ensure that the full contributions of each author are acknowledged in the "Add/Edit/Remove Authors" section of our submission form.

The list of CRediT author contributions may be found here: https://journals.plos.org/plospathogens/s/authorship#loc-author-contributions

2) We ask that a manuscript source file is provided at Revision. Please upload your manuscript file as a .doc, .docx, .rtf or .tex. If you are providing a .tex file, please upload it under the item type u2018LaTeX Source Fileu2019 and leave your .pdf version as the item type u2018Manuscriptu2019.

3) We do not publish any copyright or trademark symbols that usually accompany proprietary names, eg ©,  ®, or TM  (e.g. next to drug or reagent names). Therefore please remove all instances of trademark/copyright symbols throughout the text, including:

- TM on pages: 41, 43, 45, and 46.

4) Please upload all main figures as separate Figure files in .tif or .eps format. For more information about how to convert and format your figure files please see our guidelines:

https://journals.plos.org/plospathogens/s/figures

5) Some material included in your submission may be copyrighted. According to PLOSu2019s copyright policy, authors who use figures or other material (e.g., graphics, clipart, maps) from another author or copyright holder must demonstrate or obtain permission to publish this material under the Creative Commons Attribution 4.0 International (CC BY 4.0) License used by PLOS journals. Please closely review the details of PLOSu2019s copyright requirements here: PLOS Licenses and Copyright. If you need to request permissions from a copyright holder, you may use PLOS's Copyright Content Permission form.

Please respond directly to this email and provide any known details concerning your material's license terms and permissions required for reuse, even if you have not yet obtained copyright permissions or are unsure of your material's copyright compatibility. Once you have responded and addressed all other outstanding technical requirements, you may resubmit your manuscript within Editorial Manager.

Potential Copyright Issues:

i) Figure 1A. Please confirm whether you drew the images / clip-art within the figure panels by hand. If you did not draw the images, please provide (a) a link to the source of the images or icons and their license / terms of use; or (b) written permission from the copyright holder to publish the images or icons under our CC BY 4.0 license. Alternatively, you may replace the images with open source alternatives. See these open source resources you may use to replace images / clip-art:

- https://commons.wikimedia.org

- https://openclipart.org/.

6) When completing the data availability statement of the submission form, you indicated that you will make your data available on acceptance. We strongly recommend all authors decide on a data sharing plan before acceptance, as the process can be lengthy and hold up publication timelines. Please note that, though access restrictions are acceptable now, your entire data will need to be made freely accessible if your manuscript is accepted for publication. This policy applies to all data except where public deposition would breach compliance with the protocol approved by your research ethics board. If you are unable to adhere to our open data policy, please kindly revise your statement to explain your reasoning and we will seek the editor's input on an exemption. Please be assured that, once you have provided your new statement, the assessment of your exemption will not hold up the peer review process.

7) Please amend your detailed Financial Disclosure statement. This is published with the article. It must therefore be completed in full sentences and contain the exact wording you wish to be published.

1) State what role the funders took in the study. If the funders had no role in your study, please state: "The funders had no role in study design, data collection and analysis, decision to publish, or preparation of the manuscript."

8) Please ensure that the funders and grant numbers match between the Financial Disclosure field and the Funding Information tab in your submission form. Note that the funders must be provided in the same order in both places as well. Currently, the order of the funders is different in both places.

Note: If the reviewer comments include a recommendation to cite specific previously published works, please review and evaluate these publications to determine whether they are relevant and should be cited. There is no requirement to cite these works unless the editor has indicated otherwise.

**Reviewers' Comments:**

Reviewer's Responses to Questions

**Part I - Summary**

Please use this section to discuss strengths/weaknesses of study, novelty/significance, general execution and scholarship.

Reviewer #1: In the manuscript “A single Citrobacter rodentium infection in Pink1 knockout and wild type mice leads to regional blood-brain-barrier perturbation and glial activation without dopamine neuron axon terminal loss”, the authors investigate the potential for mild gastrointestinal infections to induce blood brain barrier disruption and inflammation in Pink1 KO and WT mice. The authors found that 26 days post Citrobacter rodentium infection led to subtle changes in BBB permeability with MRI, but did not result in infection or genotype changes in tight junction proteins. Peripheral cytokines were increased, but microglial analyses revealed only a minor increase in IBA1 protein levels and no morphological changes in striatal microglia. As the title suggests, there were no alterations detected in dopaminergic terminals, however, some of the other conclusions seem overstated for reported data. The study is well conducted and does provide valuable information on the role of Pink1 in infection, thus it could be strengthened by additional data or more careful interpretation.

Reviewer #2: Peripheral inflammation is increasingly recognized as an important factor that can influence the progression of PD. Understanding how infections and immune activation impact the brain and the BBB is therefore highly relevant.

I find the topic highly relevant and the study potentially valuable, but several aspects need revision before the conclusions can be supported. The study touches on an important aspect of PD research, namely the role of peripheral infection and inflammation in modulating brain responses. However, the manuscript requires revisions, particularly in clarifying methods, substantiating conclusions with additional data, and moderating some of the claims. As it stands, the effects on the brain are not very clear, and the tone of the discussion should be adjusted to better reflect this.

Reviewer #3: PLoS Pathogen manuscript review of 25-01667

Title: “A single Citrobacter rodentium infection in Pink1 knockout 1 and wild type mice leads to regional blood-brain-barrier perturbation and glial activation without dopamine neuron axon terminal loss”

by Mukherjee et al.

Dr. Louis Eric Trudeau and colleagues produced an interesting manuscript from their study of the effects of conditional, DAT-Cre-induced PINK1 deficiency in mice to examine a genotype-dependent difference in the outcome of a transient (single exposure-based) GI infection with a microbial pathogen, Citrobacter rodentium. The exploration of PD linked genotypic variants vs a host’s fitness against microbial threats has mushroomed in the last decade, and the Montreal team has made significant contributions to this burgeoning field, as have other groups. This manuscript follows on the heels of their published work with 4 exposure events of animals to the same pathogen (Citrobacter rodentium), which led to a transient motor phenotype seen in PINK1-deficient animals.

Dr Trudeau and his colleagues are taking on two specific themes, namely the integrity of the BBB as well as systemic vs striatal inflammation responses downstream of the chosen genotype in the context of a non-lethal, but still virulent infection.

**********

**Part II – Major Issues: Key Experiments Required for Acceptance**

Please use this section to detail the key new experiments or modifications of existing experiments that should be absolutely required to validate study conclusions.

Generally, there should be no more than 3 such required experiments or major modifications for a "Major Revision" recommendation. If more than 3 experiments are necessary to validate the study conclusions, then you are encouraged to recommend "Reject".

Reviewer #1: As noted, the title suggests glial activation is a significant outcome from infection and modulated by Pink1, however, the actual microglial activation data is quite superficial, showing only western blot increase in total Iba1, and no morphological changes in the striatum. It was not stated why CD68 was not used as a marker for microglial activation, but this is routinely used as an indicator along with morphology to determine activation status of microglia – could the authors show this as a separate analysis to understand why the western data seem to contradict the imaging? As a minor note, as astrocytes were not activated, a better description would be microglial activation and not “glial” activation when discussing this result.

Given the focus on this link to PD, it is notable that striatal terminals were the only indicator of dopaminergic dysfunction reported. For the answer if Pink1 mediated response to infection on the DA system, why were nigral DA neurons not included in any analyses? Presumably if the immune/Pink1/mitochondrial interactions that have been previously reported were involved, mitochondrial damage in DA neurons might be a predegenerative response to this type of injury. Related, could nigral microglia have a different activation status than striatum if more damage is related to intracellular changes in mitochondria in DA neurons? Likewise, neurochemistry in the striatum could have potentially been a more sensitive measure (e.g., DA and metabolite levels), than DAT and TH loss.

The MRI data demonstrate potential changes in BBB but the ZO-1 and ZO-1 western blots suggest this is not related to endothelial tight junctions. While this certainly could be true, why are other BBB proteins not measured to determine what may lead to permeability? Immunohistochemistry of the brain vasculature would be helpful to demonstrate if regional differences on MRI are related to the reported neuro-immune response and modulation by Pink1.

Reviewer #2: One of the main points that needs clarification is why the authors specifically focused on ZO-1 and ZO-2, and did not test additional tight junction or adherens proteins. Relying on just two markers feels insufficient to support the conclusion that the BBB is not compromised. Later in the study bulk transcriptomic analysis is performed on the striatum, but since this was not restricted to endothelial cells, the results may also reflect expression from other cell types such as the ventricular lining (which will be isolated with the striatum). It would strengthen the manuscript if some of the tight junction or adherens markers identified from the transcriptomic dataset could be validated at the protein level, for example by western blot or immunohistochemistry, to support the overall conclusions.

The study also addresses microglial reactivity in the striatum, using both western blot and immunofluorescence. The results are not consistent: there is an increase in Iba-1 in lysates, but no detectable difference in the tissue sections. Since Iba-1 is not specific to microglia—it is also expressed by perivascular macrophages, infiltrating monocytes, and macrophages in the choroid plexus (which could also be isolated with the striatum) —signal detected in western blots could be influenced by these populations. The flow cytometry results show that inflammatory monocytes are present, even if not significantly altered, which complicates interpretation. Because the immunohistochemistry data are spatially restricted to the striatum, I would place more weight on those results. At present, the increase in Iba-1 in lysates does not provide strong enough evidence for microglial reactivity, and the conclusions in this section should be revised accordingly.

The flow cytometry data also reveal an increase in neutrophils in the brain. However, the evidence presented does not distinguish whether these cells extravasate into the parenchyma or remain confined to the vasculature. Without histological confirmation, an increase in neutrophils by flow cytometry is not sufficient to conclude that they infiltrate the brain tissue.

Finally, I would recommend revising the discussion to tone down some of the conclusions. For instance, the statement that Citrobacter infection perturbs BBB integrity, facilitates entry of immune cells, and initiates or exacerbates PD-related pathology feels too strong based on the data presented here (lines 642 - 645). Aside from MRI, there is little direct evidence of BBB breakdown, and no histological data showing infiltration of T cells or neutrophils into the brain parenchyma. The discussion would benefit from a more cautious interpretation that reflects the limitations of the dataset. Altogether, the title should also be adjusted so that it better represents the study’s overall findings.

Reviewer #3: I welcome interrogations of host genome vs environmental threat interactions to model the pathogenesis of PD phenotypes.

My comments include the following concerns, suggestions and questions:

1. The manuscript is an assembly of many different investigative methods, which at times feels somewhat disconnected given the choice of genotype. Where does one expect a difference based on DAT-dependent change in PINK1 gene expression?

2. Related to that, the reader doesn’t easily understand why the team chose a DAT-cre-dependent cKO model for PINK1 deficiency, in particular without the authors explaining where in all their tissues DAT is expressed; by inference, when is PINK1 normally expressed in the cells of their target organs? The field has seen a very restricted and context-dependent pattern of (mitochondrial) PINK1 function;

3. Related to that, one misses the precise definition of the WT genotype. How were the animals bred, were some pairings done with heterozygotes?

4. In what context was the tomato reporter protein used, why was it never shown? It would have easily provided some answers to point #2 made above;

5. Did any adverse event / mortality occur in any infected mice?

6. Was there a sex effect in any readouts?

7. In Fig. 2, the reader can’t identify which comparisons were significantly different although the p values were listed on the side;

8. The real money one could argue lies in the transcriptome analysis given the choice in genotypic construct and comparison. Did the authors consider carrying out transcriptomics in the spleen homogenates?

9. Did the authors attempt to validate any mRNA change identified, such as by qPCR or Western blotting? I think validation steps would greatly enhance the relevance of their findings;

10. Did the authors make any comparison in their readouts and findings from the single (here) vs 4 exposure (published) events with the pathogen, whether it be in the WT or DAT-cre cKO animals, and whether it be by mRNA change, Iba1 signal change, MRI analysis or FACS analyses of the spleen? This, to answer which transient changes may be of greatest relevance as a potential biomarker when one considers a more sustained (or reoccurring) 2nd hit, ie, the C. rodentium microbe exposure;

11. I think the Discussion section should address some of the shortcomings / limitations of their study.

**********

**Part III – Minor Issues: Editorial and Data Presentation Modifications**

Please use this section for editorial suggestions as well as relatively minor modifications of existing data that would enhance clarity.

Reviewer #1: The results state that the enlargement of the spleen was higher at day 13 compared to day 26 (Line 147 – 149). These groups are not directly compared to one another, the degree of change from control to experimental groups is similar, and the raw values are similar indicating that one time point is not necessarily more severe than another. Additionally, the results are interpreted differently in the figure legend for figure 1. Restating results to be consistent with the figure legend and the data would be beneficial.

Acronyms should be defined after first use. (i.e. Line 177: contrast agent)

Figure 2 – pre to post infection vs genotype, difficult to see the change in pre vs post infection, could it be represented as percent control or normalized or put on a different scale?

Figure 11 text is too small to read – increase font size.

Reviewer #2: In light of the authors’ previous work showing that cytotoxic T cells infiltrate the brain in Pink1 knockout mice and attack dopaminergic neurons, it would be interesting to know whether this study provides any new insights into how T cells enter the brain or how they might interact with neural cells.

The transcriptome data are another valuable part of the study. It would be helpful if the authors highlighted which immune-related genes are upregulated, and whether these genes relate to the presence of neutrophils or other immune responses observed.

Another important aspect is the mouse strain background. Different strains can show very different responses to acute or chronic infection, so this information should be provided. Similarly, details on how the brains were isolated are missing. Were they perfused with saline prior to homogenization and analysis? This methodological detail can strongly influence interpretation and should be reported clearly in the methods.

Reviewer #3: n/a

**********

PLOS authors have the option to publish the peer review history of their article (what does this mean?). If published, this will include your full peer review and any attached files.

If you choose “no”, your identity will remain anonymous but your review may still be made public.

**Do you want your identity to be public for this peer review?** For information about this choice, including consent withdrawal, please see our Privacy Policy.

Reviewer #1: No

Reviewer #2: **Yes:**wouter peelaerts

Reviewer #3: **Yes:**Michael Schlossmacher

[NOTE: If reviewer comments were submitted as an attachment file, they will be attached to this email and accessible via the submission site. Please log into your account, locate the manuscript record, and check for the action link "View Attachments". If this link does not appear, there are no attachment files.]

**Figure resubmission:**

While revising your submission, we strongly recommend that you use PLOS’s NAAS tool (https://ngplosjournals.pagemajik.ai/artanalysis) to test your figure files. NAAS can convert your figure files to the TIFF file type and meet basic requirements (such as print size, resolution), or provide you with a report on issues that do not meet our requirements and that NAAS cannot fix.

After uploading your figures to PLOS’s NAAS tool - https://ngplosjournals.pagemajik.ai/artanalysis, NAAS will process the files provided and display the results in the "Uploaded Files" section of the page as the processing is complete. If the uploaded figures meet our requirements (or NAAS is able to fix the files to meet our requirements), the figure will be marked as "fixed" above. If NAAS is unable to fix the files, a red "failed" label will appear above. When NAAS has confirmed that the figure files meet our requirements, please download the file via the download option, and include these NAAS processed figure files when submitting your revised manuscript.

**Reproducibility:**

To enhance the reproducibility of your results, we recommend that authors of applicable studies deposit laboratory protocols in protocols.io, where a protocol can be assigned its own identifier (DOI) such that it can be cited independently in the future. Additionally, PLOS ONE offers an option to publish peer-reviewed clinical study protocols. Read more information on sharing protocols at https://plos.org/protocols?utm_medium=editorial-email&utm_source=authorletters&utm_campaign=protocols

# Author comment: Author response to Decision Letter 1

**DOI:** 10.1371/journal.ppat.1014315.r002

2 Feb 2026

Attachment Submitted filename: Response_reviewers_Trudeau_Plos_Pathogens.pdf

# Peer review: Decision Letter 1

**DOI:** 10.1371/journal.ppat.1014315.r003

**Authors:** D. Scott Samuels, Amanda L. Woerman

9 Mar 2026

PPATHOGENS-D-25-01667R1

A single Citrobacter rodentium infection in Pink1 knockout and wild type mice leads to regional blood-brain-barrier perturbation and limited microglial activation without dopamine neuron axon terminal loss

PLOS Pathogens

Dear Dr. Trudeau,

Thank you for submitting your manuscript to PLOS Pathogens. After careful consideration, there are two minor revisions, identified below, that should be addressed prior to acceptance. Therefore, we invite you to submit a revised version of the manuscript that addresses the points raised during the review process.

Please submit your revised manuscript by May 08 2026 11:59PM. If you will need more time than this to complete your revisions, please reply to this message or contact the journal office at plospathogens@plos.org. When you're ready to submit your revision, log on to https://www.editorialmanager.com/ppathogens/ and select the 'Submissions Needing Revision' folder to locate your manuscript file.

Please include the following items when submitting your revised manuscript:

* A letter that responds to each point raised by the editor and reviewer(s). You should upload this letter as a separate file labeled 'Response to Reviewers'. This file does not need to include responses to any formatting updates and technical items listed in the 'Journal Requirements' section below.

* A marked-up copy of your manuscript that highlights changes made to the original version. You should upload this as a separate file labeled 'Revised Manuscript with Track Changes'.

* An unmarked version of your revised paper without tracked changes. You should upload this as a separate file labeled 'Manuscript'.

If you would like to make changes to your financial disclosure, competing interests statement, or data availability statement, please make these updates within the submission form at the time of resubmission. Guidelines for resubmitting your figure files are available below the reviewer comments at the end of this letter.

We look forward to receiving your revised manuscript.

Kind regards,

Amanda L. Woerman

Academic Editor

PLOS Pathogens

D. Scott Samuels

Section Editor

PLOS Pathogens

Sumita Bhaduri-McIntosh

Editor-in-Chief

PLOS Pathogens

orcid.org/0000-0003-2946-9497

Michael Malim

Editor-in-Chief

PLOS Pathogens

orcid.org/0000-0002-7699-2064

**Additional Editor Comments:**

As identified by the Reviewers, there are two small changes that should be made to the manuscript prior to acceptance. The first is to adjust the short title such that it is consistent with the findings of the study. The second is to address the lack of sex-specific analyses in the Discussion.

**Journal Requirements:**

If the reviewer comments include a recommendation to cite specific previously published works, please review and evaluate these publications to determine whether they are relevant and should be cited. There is no requirement to cite these works unless the editor has indicated otherwise.

1) We do not publish any copyright or trademark symbols that usually accompany proprietary names, eg ©,  ®, or TM  (e.g. next to drug or reagent names). Therefore please remove all instances of trademark/copyright symbols throughout the text, including:

- TM on page: 27.

2) When completing the data availability statement of the submission form, you indicated that you will make your data available on acceptance. We strongly recommend all authors decide on a data sharing plan before acceptance, as the process can be lengthy and hold up publication timelines. Please note that, though access restrictions are acceptable now, your entire data will need to be made freely accessible if your manuscript is accepted for publication. This policy applies to all data except where public deposition would breach compliance with the protocol approved by your research ethics board. If you are unable to adhere to our open data policy, please kindly revise your statement to explain your reasoning and we will seek the editor's input on an exemption. Please be assured that, once you have provided your new statement, the assessment of your exemption will not hold up the peer review process.

3) Please make sure to include the correct citation for Biorender in the legends of Figures 1A and 13.

**Reviewers' Comments:**

Reviewer's Responses to Questions

**Part I - Summary**

Please use this section to discuss strengths/weaknesses of study, novelty/significance, general execution and scholarship.

Reviewer #1: The authors have addressed each concern and the revised manuscript represents a rigorous study.

Reviewer #2: The authors have addressed all points. A minor issue related to the short title remains to be adjusted.

Reviewer #3: The body of work contributes to the field of PD pathogenesis and neurodegeneration in two ways: exploring the interaction between genetic risk alleles and environmental exposure; and learning more about the systemic effects of PINK1, which has been understudied in its biology (other than its role in phosphorylation of ubiquitin and mitophagy).

I think the revision has been thoughtfully done, and the authors have carefully addressed the issues that issues raised by the reviewers, either with new experimental results or with a refined discussion.

My only request to the authors is to state in the Discussion section that their study was underpowered for a sex analysis (see rebuttal letter). I didnt see it there yet.

**********

**Part II – Major Issues: Key Experiments Required for Acceptance**

Please use this section to detail the key new experiments or modifications of existing experiments that should be absolutely required to validate study conclusions.

Generally, there should be no more than 3 such required experiments or major modifications for a "Major Revision" recommendation. If more than 3 experiments are necessary to validate the study conclusions, then you are encouraged to recommend "Reject".

Reviewer #1: n/a

Reviewer #2: (No Response)

Reviewer #3: n/a

**********

**Part III – Minor Issues: Editorial and Data Presentation Modifications**

Please use this section for editorial suggestions as well as relatively minor modifications of existing data that would enhance clarity.

Reviewer #1: n/a

Reviewer #2: The short title still needs to be adjusted. The short title does not reflect the changes that are requested.

The main title now more accurately reflects the findings of the study.

Main title: A single Citrobacter rodentium infection in Pink1 knockout and wild type mice leads to

regional blood-brain-barrier perturbation and limited microglial activation without

dopamine neuron axon terminal loss

Short title: Gut infection disrupts BBB and activates microglia in Pink1 KO mice

The short title is still misleading, and needs to be adjusted to better reflect the study's findings. Based on the presented data, the disruption of the BBB is very modest, and microglial activation is simply incorrect (it is absent). If there is any activation related to macrophages, then these are more likely to be vascular monocytes, but again, no real evidence is presented for this either.

Reviewer #3: n/a

**********

PLOS authors have the option to publish the peer review history of their article (what does this mean?). If published, this will include your full peer review and any attached files.

If you choose “no”, your identity will remain anonymous but your review may still be made public.

**Do you want your identity to be public for this peer review?** For information about this choice, including consent withdrawal, please see our Privacy Policy.

Reviewer #1: No

Reviewer #2: No

Reviewer #3: **Yes:**Michael G. Schlossmacher

**Figure resubmission:**

While revising your submission, we strongly recommend that you use PLOS’s NAAS tool (https://ngplosjournals.pagemajik.ai/artanalysis) to test your figure files. NAAS can convert your figure files to the TIFF file type and meet basic requirements (such as print size, resolution), or provide you with a report on issues that do not meet our requirements and that NAAS cannot fix.

After uploading your figures to PLOS’s NAAS tool - https://ngplosjournals.pagemajik.ai/artanalysis, NAAS will process the files provided and display the results in the "Uploaded Files" section of the page as the processing is complete. If the uploaded figures meet our requirements (or NAAS is able to fix the files to meet our requirements), the figure will be marked as "fixed" above. If NAAS is unable to fix the files, a red "failed" label will appear above. When NAAS has confirmed that the figure files meet our requirements, please download the file via the download option, and include these NAAS processed figure files when submitting your revised manuscript.

**Reproducibility:**

To enhance the reproducibility of your results, we recommend that authors of applicable studies deposit laboratory protocols in protocols.io, where a protocol can be assigned its own identifier (DOI) such that it can be cited independently in the future. Additionally, PLOS ONE offers an option to publish peer-reviewed clinical study protocols. Read more information on sharing protocols at https://plos.org/protocols?utm_medium=editorial-email&utm_source=authorletters&utm_campaign=protocols

# Author comment: Author response to Decision Letter 2

**DOI:** 10.1371/journal.ppat.1014315.r004

27 May 2026

Attachment Submitted filename: Response_reviewers_Trudeau_Plos_Pathogens_May15_2026.pdf

# Editor report: Decision Letter 2

**DOI:** 10.1371/journal.ppat.1014315.r005

**Authors:** D. Scott Samuels, Amanda L. Woerman

28 May 2026

Dear Dr. Trudeau,

We are pleased to inform you that your manuscript 'A single Citrobacter rodentium infection in Pink1 knockout and wild type mice leads to regional blood-brain-barrier perturbation and limited microglial activation without dopamine neuron axon terminal loss' has been provisionally accepted for publication in PLOS Pathogens.

Before your manuscript can be formally accepted you will need to complete some formatting changes, which you will receive in a follow up email. A member of our team will be in touch with a set of requests.

Please note that your manuscript will not be scheduled for publication until you have made the required changes, so a swift response is appreciated.

IMPORTANT: The editorial review process is now complete. PLOS will only permit corrections to spelling, formatting or significant scientific errors from this point onwards. Requests for major changes, or any which affect the scientific understanding of your work, will cause delays to the publication date of your manuscript.

Should you, your institution's press office or the journal office choose to press release your paper, you will automatically be opted out of early publication. We ask that you notify us now if you or your institution is planning to press release the article. All press must be co-ordinated with PLOS.

Thank you again for supporting Open Access publishing; we are looking forward to publishing your work in PLOS Pathogens.

Best regards,

Amanda L. Woerman

Academic Editor

PLOS Pathogens

D. Scott Samuels

Section Editor

PLOS Pathogens

Sumita Bhaduri-McIntosh

Editor-in-Chief

PLOS Pathogens

orcid.org/0000-0003-2946-9497

Michael Malim

Editor-in-Chief

PLOS Pathogens

orcid.org/0000-0002-7699-2064

***********************************************************

Reviewer Comments (if any, and for reference):

# Editor report: Acceptance letter

**DOI:** 10.1371/journal.ppat.1014315.r006

**Authors:** D. Scott Samuels, Amanda L. Woerman

Dear Dr. Trudeau,

We are delighted to inform you that your manuscript, "A single Citrobacter rodentium infection in Pink1 knockout and wild type mice leads to regional blood-brain-barrier perturbation and limited microglial activation without dopamine neuron axon terminal loss," has been formally accepted for publication in PLOS Pathogens.

We have now passed your article onto the PLOS Production Department who will complete the rest of the pre-publication process. All authors will receive a confirmation email upon publication.

The corresponding author will soon be receiving a typeset proof for review, to ensure errors have not been introduced during production. Please review the PDF proof of your manuscript carefully, as this is the last chance to correct any scientific or type-setting errors. Please note that major changes, or those which affect the scientific understanding of the work, will likely cause delays to the publication date of your manuscript. Note: Proofs for Front Matter articles (Pearls, Reviews, Opinions, etc...) are generated on a different schedule and may not be made available as quickly.

Soon after your final files are uploaded, the early version of your manuscript, if you opted to have an early version of your article, will be published online. The date of the early version will be your article's publication date. The final article will be published to the same URL, and all versions of the paper will be accessible to readers.

For Research Articles, you will receive an invoice from PLOS for your publication fee after your manuscript has reached the completed accept phase. If you receive an email requesting payment before acceptance or for any other service, this may be a phishing scheme. Learn how to identify phishing emails and protect your accounts at https://explore.plos.org/phishing.

Thank you again for supporting open-access publishing; we are looking forward to publishing your work in PLOS Pathogens.

Best regards,

Sumita Bhaduri-McIntosh

Editor-in-Chief

PLOS Pathogens

orcid.org/0000-0003-2946-9497

Michael Malim

Editor-in-Chief

PLOS Pathogens

orcid.org/0000-0002-7699-2064
